# Airway Management of a Patient With Penetrating Maxillofacial Trauma Caused by Chainsaw Kickback: A Case Report

**DOI:** 10.7759/cureus.45064

**Published:** 2023-09-11

**Authors:** Ryo Wakabayashi

**Affiliations:** 1 Department of Anesthesia, Nagano Red Cross Hospital, Nagano, JPN

**Keywords:** videolaryngoscope, oxygenation, maxillofacial trauma, chainsaw, awake intubation

## Abstract

Anesthesiologists rarely experience airway management in patients with maxillofacial injuries caused by a chainsaw. A 36-year-old male was referred to our hospital because of maxillofacial injuries caused by chainsaw kickback. There were deep lacerations of the right eyelid, medial canthus, cheek, and jaw with venous bleeding. The laceration of the cheek reached the oral cavity and looked like a “second mouth.” The patient was taken to the operating room for urgent laceration repair under general anesthesia. Despite a poor laryngeal view, awake orotracheal intubation with a videolaryngoscope was successful on the second attempt without complications. Oxygenation was optimized by supplemental oxygen administration via a suction catheter inserted from the “second mouth” throughout the airway management. The present case highlights the importance of airway management strategies according to the nature of the trauma in patients with penetrating maxillofacial trauma caused by a chainsaw.

## Introduction

Patients with maxillofacial trauma present serious challenges for anesthesiologists because airway management in those patients can be complicated by disarranged anatomy, soft tissue injury, edema, associated hemorrhage, and a full stomach [1-3]. However, there is no evidence-based approach regarding the best practice for airway management in patients with maxillofacial trauma [2]. Therefore, a clinical decision depends on the patient’s condition, clinical setting, injuries to the airway and other organs, and available personnel, expertise, and equipment [2].

Maxillofacial injuries are mainly caused by traffic accidents and assaults [4,5], while there have been very few reports of maxillofacial trauma caused by a chainsaw [6]. Therefore, anesthesiologists rarely experience airway management in patients with maxillofacial trauma secondary to chainsaw injuries. Herein, I report a case of successful airway management in a patient with penetrating maxillofacial trauma caused by chainsaw kickback. Written informed consent for the publication of this report was obtained from the patient.

## Case presentation

**Figure 1:**
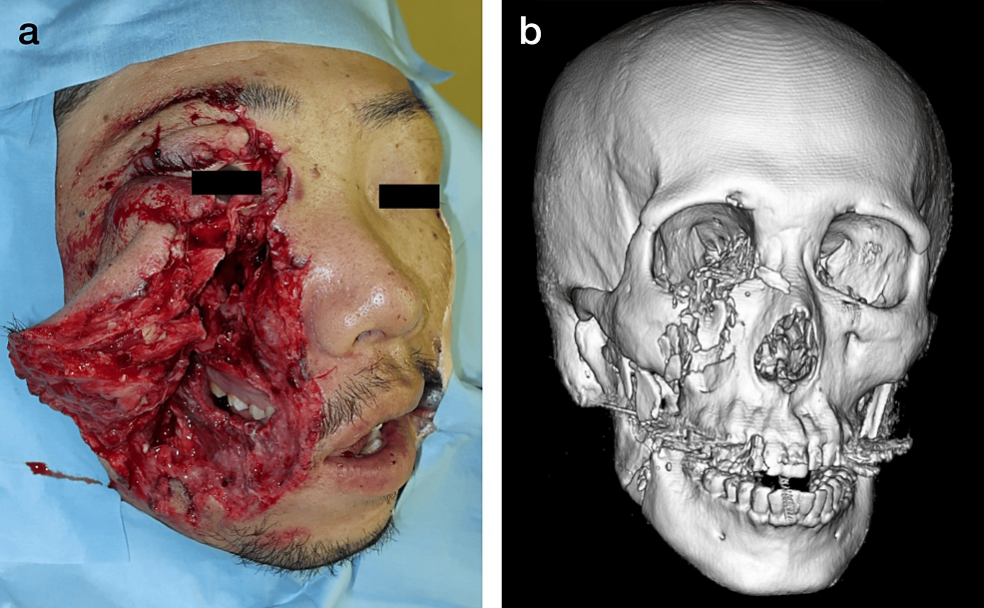
The nature of the maxillofacial trauma A clinical photograph shows deep lacerations of the right eyelid, medial canthus, cheek, and jaw with venous bleeding (a). Three-dimensional computed tomography of the head shows multiple fractures of the right frontal bone, zygomatic bone, maxilla, and mandible (b).

A 36-year-male (height: 171 cm, weight: 54 kg) with American Society of Anesthesiologists physical status classification IIIE was referred to our hospital due to maxillofacial injuries caused by chainsaw kickback. On the patient’s presentation to the hospital, his blood pressure was 100/60 mmHg, heart rate was 60 beats/minute, respiratory rate was 22 breaths/minute, and percutaneous oxygen saturation (SpO_2_) was 99% while he was breathing ambient air. There were deep lacerations of the right eyelid, medial canthus, cheek, and jaw with venous bleeding (Figure 1a). The laceration of the cheek reached the oral cavity and looked like a “second mouth.” Computed tomography of the head revealed multiple fractures of the right frontal bone, zygomatic bone, maxilla, and mandible (Figure 1b). There was no evidence of basilar skull fractures, pneumocephalus, or trauma of the cervical spine. The patient could open his mouth approximately 3 cm, although there was pain. Preoperative test results, including results of blood screening, venous blood gas analysis, chest X-ray examination, and electrocardiogram were within normal limits.

The patient was taken to the operating room for urgent laceration repair under general anesthesia. The duration of preoperative fasting was approximately six hours. The patient’s vital signs upon entering the operating room were as follows: blood pressure, 114/65 mmHg; heart rate, 57 beats/minute; respiratory rate, 20 breaths/minute; and SpO_2_, 99% at room air. Oxygenation was performed by 10 L/minute oxygen insufflation via a 14 Fr suction catheter inserted from the “second mouth” throughout the airway management. Continuous infusion of remifentanil (0.05 μg/kg/minute) was started for conscious sedation during awake intubation. Topical anesthesia of the pharyngeal and laryngeal mucosa was performed by spraying 4 mL of 4% lidocaine. Videolaryngoscopy using a McGRATH™ MAC videolaryngoscope (Medtronic, Dublin, Ireland) revealed a modified Cormack-Lehane grade Ⅱb view [7] and blood contamination of the larynx. Orotracheal intubation using a tracheal tube with an internal diameter of 8.0 mm was successful in the second attempt. The predicted effect site concentration of remifentanil was approximately 1 ng/mL [8] when the trachea was intubated. During the airway management, respiratory rate and SpO_2_ were maintained at 14-20 breaths/minute and 99%-100%, respectively, and pulmonary aspiration of gastric contents was not observed. After intubation, general anesthesia was induced with 2.8 mg/kg propofol and maintained with 2% of sevoflurane or 4% of desflurane, 0.1-0.5 μg/kg/minute of remifentanil, and 3.7 μg/kg of fentanyl. The surgery was completed uneventfully in 106 minutes. The total amount of fluid infusion was 1,750 mL, total blood loss was 20 mL, and total urine volume was 140 mL. The patient underwent open reduction and internal fixation of the right mandibular fracture on postoperative day 12 and was discharged on postoperative day 21 without any complications.

## Discussion

Safe and optimal airway management of patients with maxillofacial injuries requires an appreciation of the nature of the trauma [3]. In patients with maxillofacial trauma caused by a chainsaw, soft tissue lacerations, sometimes with severe avulsions, are most frequently observed, as well as facial bone fractures and teeth injuries [6]. The patient had deep lacerations of the right eyelid, medial canthus, cheek, and jaw with venous bleeding and multiple fractures of the right frontal bone, zygomatic bone, maxilla, and mandible. Therefore, the potential risks for airway management of the patient included (1) difficult intubation due to bleeding, (2) difficult ventilation because of the penetrating maxillofacial trauma, and (3) uncertain safety of nasal oxygenation and nasotracheal intubation. According to these considerations, the strategy of airway management was performed as described above.

Since difficult intubation and ventilation were expected, awake intubation was chosen [9,10]. Recent guidelines for difficult airway management recommend awake intubation in patients with anticipated difficult airways because of its high success rate of 88%-100% and favorable safety profile provided by maintained spontaneous ventilation and intrinsic airway tone [8,9]. Considering the uncertain safety of nasotracheal intubation [2] and the absence of limited mouth opening, orotracheal intubation was performed in the present case. Although mandibular and zygomatic arch injuries can cause trismus [2], this patient did not have trismus. A systematic review and meta-analysis has shown that awake intubation using videolaryngoscopy has a success rate and safety profile comparable to those using flexible bronchoscopy [11]. In patients with airway bleeding, videolaryngoscopy could be the first choice for awake intubation [10]. In this patient, despite the poor laryngeal view, orotracheal intubation with a McGRATH™ MAC videolaryngoscope was successful on the second attempt. Remifentanil is used for conscious sedation during awake intubation at an effect site concentration of 1-3 ng/mL [8]. Remifentanil is associated with high levels of patient satisfaction and low risk of over-sedation and airway obstruction when used for awake intubation [12]. This patient did not present remifentanil-related adverse events during airway management at the predicted effect site concentration of remifentanil of approximately 1 ng/mL [8].

Optimizing oxygenation is of paramount importance in airway management. Recent guidelines for difficult airway management recommend supplemental oxygen administration throughout awake intubation [9,10]. During awake orotracheal intubation, supplemental oxygenation is commonly performed via a low- or high-flow nasal cannula [9,10]. However, in this patient, oxygen administration via a nasal cannula was not performed because of his severe facial injuries [2,10], although no basilar skull fractures were shown in the preoperative computed tomography. Oxygen administration (10 L/minute) was thus performed via a suction catheter inserted from the “second mouth.” The incidence of desaturation (SpO_2_ ≤ 90%) with low-flow (<30 L/minute) oxygen administration during awake intubation ranges from 12% to 16% [13-15]. In the present case, however, SpO_2_ was maintained at 99%-100% throughout the airway management. Therefore, oxygen insufflation from the “second mouth” might have been effective as an alternative to nasal oxygenation.

## Conclusions

A case of successful airway management in a patient with penetrating maxillofacial trauma caused by chainsaw kickback was presented. The present case indicates the importance of airway management strategies according to the nature of the trauma in patients with penetrating maxillofacial trauma.
